# Distal Femur Valgus Deformity After Rigid Intramedullary Nailing of Adolescent Femoral Shaft Fracture

**DOI:** 10.5435/JAAOSGlobal-D-22-00220

**Published:** 2023-10-06

**Authors:** Michael G. Burton, John Y. Moon, David W. Roberts

**Affiliations:** From the University of Illinois College of Medicine, Department of Orthopaedic Surgery, Chicago, IL (Dr. Burton and Dr. Moon); Department of Pediatric Orthopaedic Surgery and Scoliosis, Northshore University Health System, Chicago, IL (Dr. Roberts).

## Abstract

A 12-year-old girl developed a distal femoral shaft fracture treated with lateral trochanteric entry intramedullary nail fixation. The nail was retained after union because of a persistent nonossifying fibroma at the previous fracture site. At 16 months after surgery, marked valgus deformity was noted at the distal femur, with signs of implant haloing and loosening, suggesting repetitive motion and stress concentration of forces at the distal femur. Owing to recognition before skeletal maturity, the valgus was corrected with hemiepiphysiodesis. This finding illustrates the importance of follow-up up to skeletal maturity for pediatric femoral shaft fractures and consideration of routine removal of implants after fracture union to avoid this previously unreported complication.

## Background

Femoral shaft fractures account for approximately 2% of all pediatric fractures.^1^ Older children with femoral shaft fractures are commonly treated surgically, and the optimal choice of fixation remains controversial—depending on fracture pattern, patient weight, and surgeon preference. An elastic stable intramedullary nail (ESIN) is most used in children younger than 10 years, with high rates of union and low risk of complications.^2^ However, shortening and malunion may occur in length-unstable fracture patterns, heavy patients, or older patients.^3,4^ For these patients, rigid fixation methods are preferred—either with submuscular plate fixation or lateral-entry trochanteric rigid intramedullary nail (IMN) fixation.

Submuscular plate fixation uses a minimally invasive technique to insert a plate across the fracture site in a submuscular tunnel, which is then fixed proximally and distally to create a bridging construct. Results are favorable with high union rates and low risk of complications.^5,6^

Distal femoral valgus deformity (DFVD) has been described as a unique complication of pediatric femoral shaft fractures treated with submuscular bridge plating.^7-9^ The mechanism is unclear, but has been hypothesized to relate to distal positioning or contouring of the plate. The distance of the plate and proximity of the fracture in relation to the physis have also been suggested as possible factors.^9^ Some patients with DFVD developed notable deformity and required osteotomy for correction.^8,9^ Routine implant removal after fracture healing has been recommended to avoid this complication.

With the development of lateral-entry trochanteric implants, rigid IMN fixation has been increasingly used for adolescent femoral shaft fractures. Results are excellent with early mobilization, high union rates, and low rates of complications. The lateral trochanteric entry point avoids the femoral head blood supply and seems to avoid the risk of osteonecrosis. One study did show some patients to have developed valgus deformity of the proximal femur, but this was mild and did not require treatment.^10^ Other reports using lateral-entry IMNs have not reported this complication.^11,12^

DFVD has not been reported in the literature after rigid IMN fixation of pediatric femoral shaft fractures. This is the first case report that profiles a patient who developed DFVD after treatment of a femoral shaft fracture with rigid IMN fixation, which was corrected with hemiepiphysiodesis. This emphasizes the importance of monitoring for DFVD after femoral shaft fracture in skeletally immature patients.

## Case Summary

**Figure 1:**
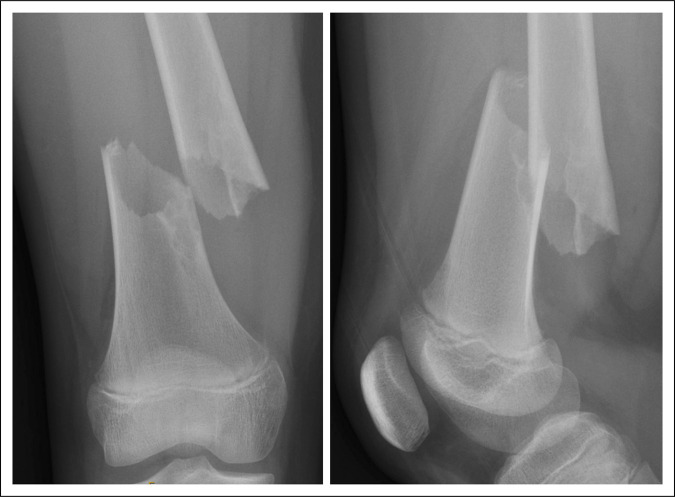
Preoperative radiographs of a displaced distal third femoral shaft fracture through a nonossifying fibroma in a skeletally immature patient.

A 12-year-old girl presented to the emergency department with a closed right femoral shaft fracture after her sled collided with a tree. Radiographs showed a displaced distal third femoral shaft fracture through an eccentric bubbly lesion consistent with a nonossifying fibroma (Figure 1).

**Figure 2:**
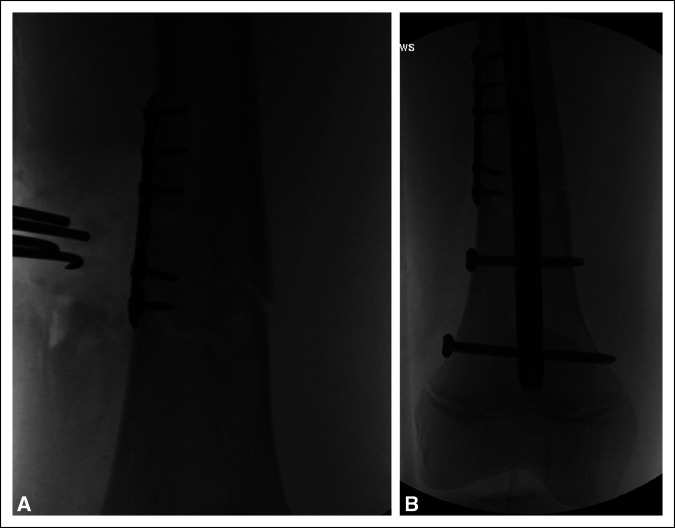
Radiographs showing A**,** anatomic reduction achieved with unicortical screws and plate and **B,** definitive fixation after a rigid intramedullary nail was inserted.

Treatment was recommended with a lateral trochanteric entry rigid IMN. During surgery, open reduction was needed to restore fracture alignment, and reduction was held during nailing with a small unicortical plate to maintain reduction during reaming and IMN placement, a useful technique described for maintaining alignment during IMN fixation of metaphyseal fractures.^13,14^ Anatomic reduction was obtained (Figure 2).

**Figure 3:**
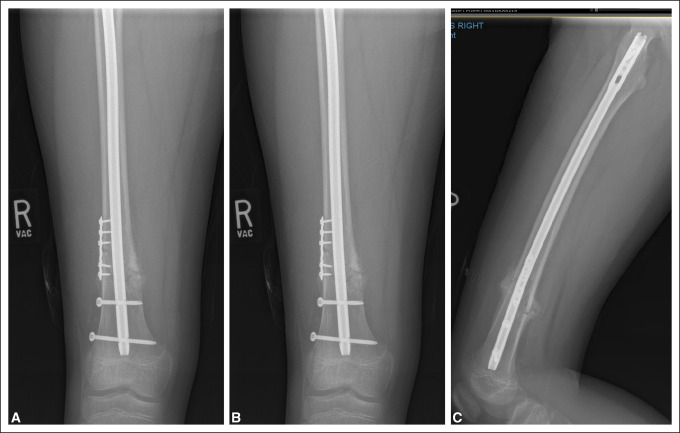
Radiographs obtained 6 weeks postoperatively demonstrating healed femoral shaft fracture with rigid intramedullary nail fixation.

**Figure 4:**
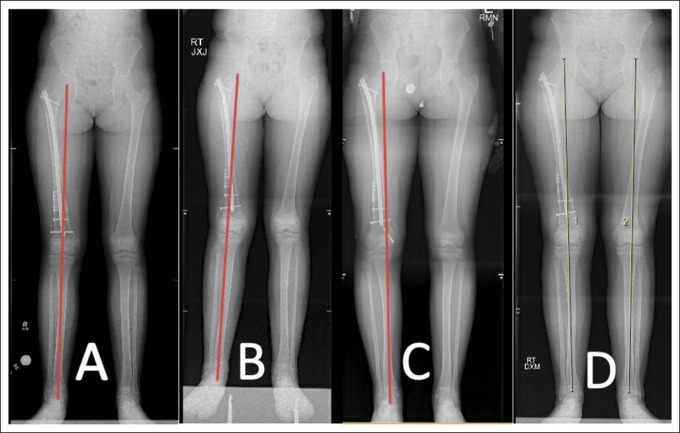
**A,** Initial postoperative radiograph with a neutral mechanical axis. **B,** Radiograph showing right distal femoral valgus noted 16 months after the index procedure. **C,** Radiograph showing correction with medial hemiepiphysiodesis of the distal femur, obtained 3 months postoperatively. **D,** Radiograph showing the maintained mechanical axis 1 year later. Transphyseal screw removed.

**Figure 5:**
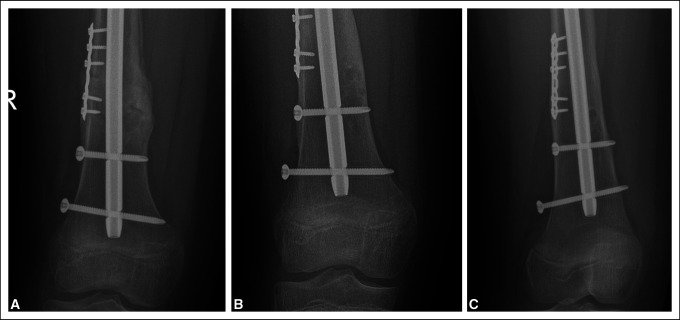
Radiographs showing evidence of implant loosening with haloing visualized around the distal tip of the nail and locking bolt. **A,** Not seen at 3 months. **B,** First noted at 7 months postoperatively with mild haloing around distal interlock. **C,** Valgus deformity seen in conjunction with a loosened distal interlocking screw.

Early weight-bearing and mobilization was permitted, and the fracture healed uneventfully (Figure 3). At 6-month follow-up, full-length lower extremity radiographs showed symmetric alignment and limb lengths (Figure 4A). However, at subsequent follow-up 16 months after injury, the patient was noted to have symptomatic valgus deformity, causing pain and altered gait. Interval development of DFVD was noted at the distal femur (Figure 4B). Haloing was noted around the distal IMN tip and distal interlocking bolt, suggestive of mechanical loosening from repetitive micromotion (Figure 5). Inflammatory markers obtained were negative for infection.

The patient was treated with temporary hemiepiphysiodesis with placement of a percutaneous transphyseal screw at the medial distal femur (Figure 4C). At the time of the revision procedure, cultures obtained were negative. The alignment corrected, and the transphyseal screw was removed approximately 6 months later. The IMN implant was retained because of the persistence of the nonossifying fibroma at the previous fracture site. Follow-up radiographs showed maintenance of neutral alignment with symmetric limb lengths at skeletal maturity (Figure 4D).

**Figure 6:**
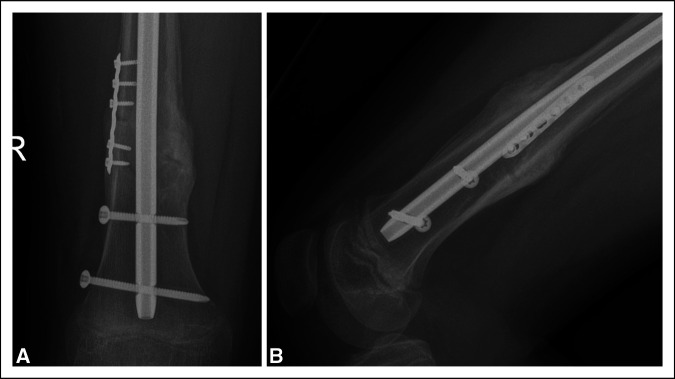
Robust bridging fracture callus seen in AP (**A**) and lateral (**B**) radiographs of the distal femur, suggestive of secondary healing of the fracture.

This case is the first reported occurrence of DFVD with a rigid IMN implant. Owing to reported cases of DFVD after submuscular plate fixation of femoral shaft fracture, routine implant has been recommended.^8,9^ The mechanism of DFVD is unknown, and various hypotheses have been suggested: increased blood flow from fracture healing with a distal location of the fracture in relation to the physis or periosteal elevation from the injury or fracture exposure during surgery. This case suggests a possible role to mechanical effects of a retained rigid femur implant, given the mechanical loosening seen at the distal interlock screw and nail tip, despite a solidly united fracture (Figure 6).

A long bone functionally acts as a beam, undergoing stress deformation with physiologic loads. We hypothesize that altering these biomechanics with a rigid implant, either IMN or submuscular plate, could alter stress and strain loads seen at parts of the bone not fixed by the implant, such as the physis. According to the Hueter-Volkmann principle, the increased stress and strain experienced by the physis distal to the rigid nail could lead to disproportionate loading of the lateral physis, or unloading of the medial physis, leading to DFVD. Stress concentration at the physis could be a plausible mechanism for posttraumatic angular deformity seen after both submuscular plating and rigid IMN fixation, but, to our knowledge, has not been reported with more flexible ESIN fixation or conservative treatment of femoral shaft fractures. Additional study of the biomechanical effects of retained implants on the physis is warranted.

This case emphasizes the importance of regular monitoring for posttraumatic deformity after treatment of pediatric femoral shaft fractures. In this case, early detection of valgus deformity while the patient was skeletally immature allowed simple treatment with hemiepiphysiodesis, avoiding the need for osteotomy as required for later diagnosis in other reports.^8,9^

## Conclusion

Femoral shaft fractures in older children are commonly treated with rigid fixation methods—either submuscular plates or lateral trochanteric entry rigid IMNs. This is the first report of DFVD after treatment of a femoral shaft fracture with a rigid IMN. In this case, early detection allowed for successful correction with hemiepiphysiodesis, with excellent clinical results at skeletal maturity.

We hypothesize that rigid implants may alter mechanical stresses seen by the distal femoral physis, which could explain why this complication has been described with rigid implants, such as submuscular plate or rigid IMN, but not flexible implants, such as ESIN. Additional study of the biomechanical effects of retained implants on the physis in skeletally immature patients is needed.

To potentially avoid this complication, we recommend routine removal of rigid IMN implants after fracture union in skeletally immature patients, as is also recommended for submuscular plates.
